# Long-term integrative personalized medicine care in end-stage renal disease and stroke: A CARE-compliant case report

**DOI:** 10.1097/MD.0000000000043541

**Published:** 2025-08-01

**Authors:** Chae-Yeon Kang, Ju-Yeon Lim, Eui-Ju Lee, Yu-Na Lee, Won-Hee Choi, Hyeon-Ji Yu

**Affiliations:** a College of Korean Medicine, Kyung Hee University, Seoul, Republic of Korea; b Department of Sasang Constitutional Medicine, Kyung Hee University College of Korean Medicine, Kyung Hee University Korean Medicine Hospital, Seoul, Republic of Korea.

**Keywords:** ESRD, integrative personalized medicine care, long-term herbal medicine, safety

## Abstract

**Rationale:**

End-stage renal disease (ESRD) is characterized by severely impaired renal function that necessitates dialysis or kidney transplantation for patient survival. Integrative Personalized Medicine Care (IPMC), which integrates conventional therapies with Traditional Korean Medicine, has been applied as a complementary approach to address ESRD and its associated complications through a holistic and individualized care model. To our knowledge, this is among the first documented cases evaluating the long-term safety and efficacy of IPMC in a patient with ESRD complicated by intracerebral hemorrhage (ICH).

**Patient concerns:**

An 80-year-old male patient undergoing dialysis for ESRD since 2021 experienced a left ICH in September 2022. Poststroke, he presented with clinical challenges including uncontrolled hypertension, nutritional imbalance, and persistent constipation.

**Diagnoses:**

The patient was diagnosed with ESRD requiring regular dialysis and later suffered from a left ICH, which further complicated his clinical condition.

**Interventions:**

The patient received IPMC at Kyung Hee University Hospital, integrating conventional treatments with individualized herbal medicine and acupuncture. Herbal prescriptions were continuously adjusted in response to clinical status, laboratory markers, and interactions with concurrent medications.

**Outcomes:**

Renal function parameters stabilized during the treatment period, and clinical control of comorbidities improved. Nutritional status improved, blood pressure was effectively managed, and symptoms of constipation were resolved. Subjective enhancements in quality of life were also reported. Importantly, no hepatic or renal toxicity was observed despite the long-term administration of herbal medicines, indicating favorable safety under medical supervision.

**Lessons:**

This case demonstrates the clinical feasibility and safety of IPMC in managing ESRD patients with concurrent complications such as ICH. Long-term integration of Traditional Korean Medicine and conventional therapies may provide adjunctive clinical benefits without compromising safety. IPMC may serve as a viable supportive strategy for addressing multifactorial challenges in ESRD management. Further large-scale cohort studies are warranted to strengthen the evidence base for this integrative treatment model.

## 1. Introduction

End-stage renal disease (ESRD) is the final stage of chronic kidney disease (CKD), characterized by a reduction in the estimated Glomerular Filtration Rate (eGFR) to <15 mL/min/1.73 m^2^.^[1]^ According to the 2024 Kidney Disease: Improving Global Outcomes (KDIGO) Guidelines, ESRD patients require life-sustaining treatments such as hemodialysis or kidney transplantation to manage complications like bone disease.

Recent studies have reported positive effects of integrating traditional Chinese medicine (TCM) with conventional treatments to improve renal function and alleviate symptoms in ESRD patients. For instance, randomized controlled trials by Li et al.^[2]^ in 2017 and Mao et al.^[3]^ in 2021 demonstrated improvements in eGFR rates in stages 3 to 4 CKD patients when TCM herbal therapy was combined with conventional treatments. Integrative medicine, which incorporates traditional and complementary alternative medicine into conventional medical interventions, adopts a holistic approach to patient care.^[4]^ Within this framework, integrative personalized medicine care (IPMC) utilizes treatment strategies tailored to the genetic and biological characteristics of individual patients.^[5]^

A meta-analysis published in 2024^[6]^ summarized evidence showing that TCM treatment effectively reduced serum creatinine levels and increased eGFR in CKD patients. Additionally, Chen cohort study in 2019^[7]^ reported lower rates of ESRD progression and mortality in diabetic nephropathy patients who used TCM compared to those who did not. These findings collectively suggest that personalized integration of TCM into standard CKD management can effectively slow disease progression and improve patient outcomes.

Therefore, our team has administered personalized herbal prescriptions alongside conventional treatment to a ESRD patient over a prolonged period. By closely monitoring the patient’s condition in a hospital setting and managing potential interactions between herbal medicine and other drugs, we observed that IPMC administration of herbal medicine is safe and effective in improving renal function, with long-term use also demonstrating sustained safety. This report aims to discuss these findings and explain the mechanisms supporting the safety and efficacy of long-term integrative administration of herbal medicine in ESRD patients.

## 2. Case presentation

### 2.1. Patient information

The patient is an 80-year-old male, 170 cm tall and weighing 45.4 kg, who was diagnosed with CKD in 2021 and has been undergoing dialysis since August 2022. In September 2022, he experienced a left-sided intracerebral hemorrhage (ICH). To address constipation and general discomfort associated with dialysis and to rehabilitate hemiparesis due to ICH, he was admitted to the Sasang Constitutional Medicine Department at Kyung Hee University Korean Medicine Hospital from January 5, 2023, to April 29, 2024. The patient has a history of hypertension, managed with oral antihypertensive therapy since 2007, and a history of aortic dissection in 2012. In September 2022, he presented to the emergency room with right hemiparesis, and brain computed tomography confirmed a left-sided ICH, for which he has been receiving conservative treatment. His medical history also includes active tuberculosis, diagnosed in 2022, and vancomycin-resistance enterococci positivity.

At the time of admission on January 5, 2023, his daily urine output was approximately 10 mL, and he complained of dysuria, characterized by an inability to urinate despite feeling the urge. He also reported right hemiparesis, with his right upper extremity capable of resisting moderate force at 60 degrees of elevation and his right lower extremity able to resist weak force and maintain elevation for 5 seconds at 60 degrees. During his hospital stay, he experienced intermittent infection-related symptoms in May, July, August, and September 2023. There were no notable family history findings. The patient does not consume alcohol but has a 150-pack-year smoking history (3 packs/year for 50 years).

After the admission to the Sasang Constitutional Medicine Department on January 5, 2023, the patient was temporarily transferred to general surgery (GS) from March 9 to March 17, 2023, for a brachioaxillary ateriovenous graft (AVG) operation. Postoperatively, he developed edema and pain at the right AVG operation site, requiring another transfer to GS from April 13 to May 16, 2023, for inpatient treatment of AVG site infection. Following this period, he remained in the Sasang Constitutional Medicine Department until his discharge on April 29, 2024, where he received conventional treatment along with personalized medical care, prescriptions of Hyeongbangjihwang-tang (HJT), Cheonghyeol-dan (CHD), Jayun-tang (JYT) and acupuncture treatment.

This study was conducted as a retrospective observational analysis. Due to the inherent nature of its design, which involved the review of medical records, including prescription data and clinicians’ progress notes during the patient’s hospitalization, obtaining informed consent from the subject was deemed impracticable. Moreover, considering the minimal risk posed to the subject and the absence of any reasonable grounds to assume objection to participation, the requirement for informed consent was waived. This study protocol was reviewed and approved by the Institutional Review Board (IRB) of Kyung Hee University Korean Medicine Hospital (IRB No. KOMCIRB 2024-10-020, Date of Approval: November 25, 2024).

### 2.2. Diagnostic assessment

Although records from the initial diagnosis of CKD in 2021 were unavailable, serum chemistry tests conducted on January 1, 2023 showed a serum creatinine level of 7.48 mg/dL, chronic kidney disease epidemiology collaboration-estimated glomerular filtrate rate (CKD-EPI-eGFR) of 6.2129 mL/min/1.73 m^2^, and modification of diet in renal disease (MDRD)-eGFR of 7.0506 mL/min/1.73 m^2^, leading to a diagnosis of Stage 5 CKD, requiring dialysis.

Upon admission, a whole blood test performed on January 6, 2023 revealed anemia, evidenced by reduced red blood cell count (3.19 × 10^6^/µL), hemoglobin (10.7 g/dL), and hematocrit (33.5%). There were findings of elevated mean corpuscular volume (105.1 fL) and mean corpuscular hemoglobin (MCH) (33.6 pg), along with decreased mean corpuscular hemoglobin concentration (MCHC) (32.0 g/dL). Serum biochemistry showed hypoproteinemia (total protein 6.2 g/dL) and hypoalbuminemia (3.3 g/dL), indicative of malnutrition. Mild elevations in amylase (140 U/L) and lipase (195 U/L) were observed, but imaging did not reveal any signs of pancreatitis, suggesting CKD-related hyperamylasemia without pancreatitis.^[8]^

Cardiac laboratory results demonstrated elevated creatine kinase-MB (CK-MB) (2.8 ng/mL), Troponin I (637.1 pg/mL), myoglobin (285.7 ng/dL), and brain natriuretic peptide (77 pg/mL). Transthoracic echocardiography (TTE) indicated grade 1 diastolic dysfunction, and cardiac markers showed a decreasing trend over time. Acute intervention was not required based on TTE findings, and blood pressure was managed with the patient’s existing antihypertensive regimen.

A brain computed tomography scan performed on September 25, 2022, revealed left basal ganglia hemorrhage and mild to moderate cerebral cortical atrophy.

Upon admission, the patient presented with right hemiparesis and mild cognitive impairment due to intracerebral hemorrhage. To assess this condition, physical examinations were conducted throughout the hospitalization period. Modified Barthel Index conducted on January 1, 2023 scored 20, and the manual functional test yielded 22/32 for the right side and 24/32 for the left side. The Fugl-Meyer Assessment of Motor Function scored 44 for the right upper extremity, 59 for the left upper extremity, 23 for the right lower extremity, and 20 for the left lower extremity, with a total score of 67 for the right side and 79 for the left side, confirming right hemiparesis. The Berg Balance Scale score was 5/56, indicating a high risk of falls. The Global Deterioration Scale score was 3, suggesting mild cognitive impairment.

### 2.3. Diagnostic methods

**Figure 1.**
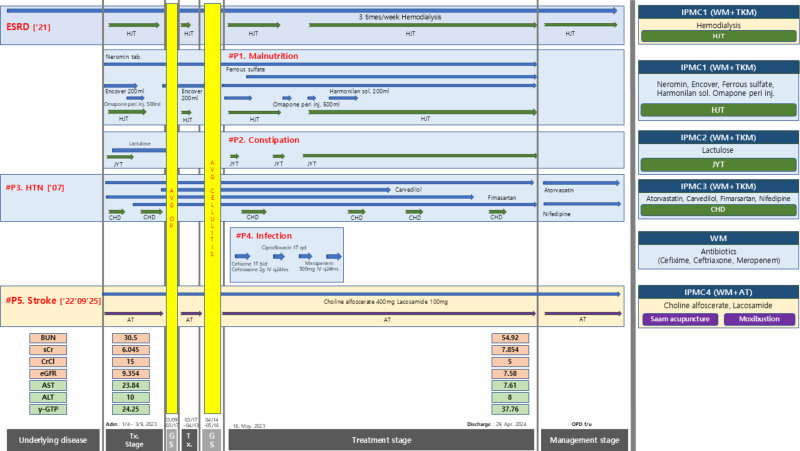
Progress of the patient’s management. The values of BUN, sCr, CrCl, eGFR, AST, ALT, δ-GTP are average of the first month upon admission (January, 2023) and the last month before discharge (April, 2024). δ-GTP = δ-glutamyl transferase; ALT = alanine transaminase; AST = aspartate aminotransferase; AT = acupuncture treatment; BUN = blood urea nitrogen; CHD = Cheonghyeol-dan; CrCl = creatinine clearance; eGFR = estimated glomerular filtration rate; ESRD = end-stage renal disease; GS = general surgery; HJT = Hyeongbangjihwang-tang; HTN = hypertension; IPMC = integrative personalized medicine care; JYT = Jayun-tang; sCr = serum creatinine; TKM = traditional Korean medicine; WM = Western Medicine

Figure 1 provides an overview of the diagnostic framework and study timeline, summarizing the methodologies employed. To assess the safety of long-term IPMC, administered for over 1 year in this ESRD patient, kidney function tests and liver function tests were conducted. Kidney function was monitored through creatinine clearance (CrCl), serum creatinine (sCr), blood urea nitrogen (BUN), and eGFR, while liver function was tracked by measuring aspartate transaminase (AST), alanine transaminase (ALT), and gamma-glutamyl transpeptidase (γ-GTP) levels.

To evaluate the efficacy of long-term IPMC, administered for over 1 year in this ESRD patient, complications of CKD and stroke were observed. Four key categories of ESRD complications were tracked: malnutrition, constipation, hypertension, and infection. For malnutrition, parameters such as albumin, body weight, hemoglobin, and calcium levels were measured. For constipation, the Bristol Stool Scale, weekly stool volume, and frequency were evaluated. The Bristol Stool Scale categorizes stool into 7 types,^[9]^ serving as an assessment tool for conditions related to the colon and stool, including irritable bowel syndrome.^[10]^ Types 1 to 2 indicate constipation, types 3 to 4 represent ideal stool, and types 6 to 7 suggest diarrhea.^[11]^ For hypertension, blood pressure was checked every Monday, using the first reading of the day before hemodialysis. Infection status was evaluated by monitoring C-reactive protein (CRP) and white blood cell (WBC) levels.

Regarding stroke, the improvement in right hemiparesis due to left ICH was evaluated by performing the Berg Balance Scale test 4 times during the hospital stay. The Berg Balance Scale is a functional balance assessment tool, known for its high validity and reliability in conditions such as Parkinson disease, multiple sclerosis, traumatic brain injury, and stroke.^[12]^ It evaluates performance across 14 tasks related to dynamic and static balance, assessing musculoskeletal, cognitive, and somatosensory abilities.^[13]^

The patient refused urinary catheterization, which precluded urine culture testing during the hospital stay. There were no other financial, cultural, or language challenges encountered.

### 2.4. Integrative personalized medicine care

#### 2.4.1. Conventional treatment

##### 2.4.1.1. IPMC1

The patient has been undergoing dialysis for ESRD since Aug, 2022. During the study period, in collaboration with nephrologists and surgeons, the patient underwent brachioaxillary AVG on Mar 15, 2023, followed by hemodialysis thrice weekly throughout the hospitalization.

For the management of malnutrition, nutritional supplements Encover (containing Biotin and 32 other ingredients) were prescribed at a dosage of 200 ml once daily from Jan 31, 2023, to Feb 6, 2023, and 600 ml once daily from Mar 9, 2023, to Apr 29, 2023. Harmonilan solution (containing ascorbic acid and 23 other ingredients) was administered at a dosage of 600 ml once daily from Apr 28, 2023, to Dec 13, 2023, followed by 200 ml once daily from Dec 20, 2023, to Jan 30, 2024, 400 ml once daily from Jan 31, 2024, to Feb 9, 2024, and 200 ml once daily again from Feb 10, 2024, to Feb 29, 2024. Additionally, Feroba-You slow-release tablets (Ferrous sulfate) were prescribed according to the patient’s condition at a dosage of 2 tablets (T) or 4 T from Jun 7, 2023, to Apr 29, 2024. Neromin tablets (containing pyridoxine and 8 other components) were administered at a dosage of 1 T or 2 T from Jan 4, 2023, to Apr 29, 2024. Omapone peri injections (TPN) were prescribed at a dosage of 500 ml daily from Jan 27, 2023, to Feb 15, 2023, from May 16, 2023, to Jun 14, 2023, from Jul 20, 2023, to Jul 26, 2023, from Aug 16, 2023, to Aug 25, 2023, and from Sep 10, 2023, to Oct 11, 2023. From May 1, 2023, to Apr 17, 2024, EpoKine prefilled injection 2000 IU (Erythropoietin) was prescribed as needed based on the patient’s condition, with no history of blood transfusions. Poshuin and Nephro tablets (Calcium acetate) were prescribed from Apr 10, 2023, until discharge, with doses adjusted according to the patient’s condition.

##### 2.4.1.2. IPMC2

For the management of constipation, Dulackhan-Easy syrup (Lactulose) was administered at a dosage of 30 ml once daily from Jan 4, 2023, to Jan 17, 2023, increased to 60 ml once daily from Jan 18, 2023, to Jan 24, 2023, then reduced to 30 ml once daily from Jan 25, 2023, to Feb 14, 2023, and finally to 15 ml once daily from Feb 15, 2023, to Mar 21, 2023, reflecting symptomatic improvement.

##### 2.4.1.3. IPMC3

For the management of hypertension, Norvasc tablets (Amlodipine) were prescribed at a dosage of 5 mg once daily from Jan 9, 2023, to Jan 15, 2023, and from Jan 18, 2023, to Jan 25, 2023. A dosage of 10 mg once daily was administered from Jan 16, 2023, to Jan 17, 2023, and again from Jan 23, 2023, to Feb 4, 2023. Dilatrend tablets (Carvedilol) were prescribed at a dosage of 25 mg once daily from Mar 4, 2023, to Nov 15, 2023. Kanarb tablets (Fimasartan) were administered at a dosage of 120 mg once daily from Jan 30, 2023, to Feb 12, 2023. Nifedix ER tablets (Nifedipine) were prescribed at a dosage of 60 mg once daily from Jan 30, 2023, to Feb 22, 2023, and then increased to 120 mg once daily until discharge.

##### 2.4.1.4. WM

To manage infection, Suprax capsules (Cefixime) were prescribed at a dose of 100 mg on May 22, 2023, and 200 mg on May 23, 2023. Meropenem injections (Meropenem) were administered at a dose of 500 mg daily from May 25, 2023, to Jun 8, 2023, and again from Aug 13, 2023, to Aug 23, 2023. Additionally, Ceftriaxone injections (Ceftriaxone) were prescribed at a dose of 4 g daily from May 23, 2023, to May 26, 2023.

##### 2.4.1.5. IPMC4

For stroke management, a collaborative approach with the neurology department was implemented. Gliatamin soft capsules (Choline alfoscerate) and Vimsk tablets (Lacosamide) were prescribed at daily doses of 800 mg and 100 mg, respectively, throughout the entire hospitalization period. Also, physical therapy was implemented, comprising 30 minutes of rehabilitative development therapy for disorders of the central nervous system and 30 minutes of gait training.

#### 2.4.2. Personalized acupuncture and moxibustion therapies

We selected an acupuncture regimen grounded in traditional acupuncture theory of meridians. To address stroke management, acupuncture was administered at the “seven acupoints for stroke,” comprising GV20, GB7, LI15, GB31, LI11, GB39, and ST36, which has been extensively investigated for their therapeutic efficacy in cerebrovascular disorders.^[14,15]^ Additionally, based on patient’s So-Yang constitutional type, we applied Saam acupuncture on LU8, KI7, SP3, and KI3 (bilaterally), aiming to strengthen kidney function and address stroke.^[16]^ A total of 21 acupoints were used per session. The patient received daily 20-minute acupuncture sessions for the first 10 days after admission and continued treatment throughout the hospitalization.

All procedures were performed by a skilled traditional Korean medicine doctor using single-use stainless steel needles (Dongbang Acupuncture Inc., Seongnam, Korea, 0.20 mm × 30 mm) inserted to a depth of 10 to 20 mm. The needles were retained for 20 minutes, with electrical stimulation at 2 Hz applied to the LI4, LI11, LR3, and ST36 acupoints. Detailed information on the acupuncture regimen is summarized in Table 1, following the STRICTA guidelines.

**Table 1:** Acupuncture therapy administered for the CKD patient.

1. Details of needling (1) Number of needle insertions per subject per session: 20 (2) Based on the 7 acupoints for stroke and kidney-tonification of Saam acupuncture, we administered acupuncture according to the patient’s past history of stroke and constitutional type, that is, So-Yang type Names of acupoints used: Baihui (GV20), Qubin (GB7), Jianyu (LI15), Fengshi (GB31), Quchi (LI11), Xuanzhong (GB39), Zusanli (ST36), Jingqu (LU8), Fuliu (KI7), Taibai (SP3), and Taixi (KI3) on both sides (3) Depth of insertion: 10–20 mm (4) Response sought: Simple insertion (5) Needle stimulation: Manual (6) Needle retention time: 20 minutes (7) Needle type: Single-use acupuncture needles (0.20 mm × 30 mm stainless steel) 2. Treatment regimen (1) Number of treatment sessions: 480 (2) Frequency and duration of treatment sessions: Once a day during the hospitalization 3. Other components of treatment (1) Details of other interventions: Electrical stimulation at 2 Hz was applied to both Hegu (LI4), Quchi (LI11), Taichong (LR3), and Zusanli (ST36) for 20 minutes. Electrical moxibustion therapy was performed on Guanyuan (CV4) and both Tianshu (ST25) for 20 minutes daily (2) Setting and context of treatment: Kyung Hee University Korean Medicine Hospital 4. Practitioner background (1) Description of participating acupuncturists: a TKM licensed doctor 5. Control or comparator interventions Not applicable

CV = conception vessel, GB = gall bladder, GV = governor vessel, KI = kidney, LI = large intestine, LR = liver, LU = lung, SP = spleen, ST = stomach, TKM = traditional Korean medicine.

In conjunction with acupuncture, moxibustion therapy was administered using ignited mugwort fibers on CV4 and bilateral ST25, providing a warm moxibustion treatment, commonly referred to as meridian heat therapy.

#### 2.4.3. Personalized herbal medicine therapies

During the treatment period, a total of 6 herbal medicines were used, categorized as main and auxiliary drugs. Personalized herbal medicines were administered as the main treatment throughout the pre-hospitalization period, considering the overall condition of the patient, while 2 auxiliary drugs were intermittently used for symptoms of hypertension (HTN) and constipation.

For ESRD patients, long-term IPMC1 was administered using Hyeongbangjihwang-tang (HJT) for over a year, with modifications made according to the patient’s condition. The standard HJT prescription (from Jan 6, 2023, to Feb 23, 2023, and again from May 16, 2023, to Jul 10, 2023.) and its variants, HJT-A (from Aug 08, 2023, to Apr 29, 2024.) and HJT-B (from Feb 22, 2023, to Apr 03, 2023.) were used to manage the patient’s ESRD along with malnutrition and edema. During the treatment period, the patient was prescribed 2 doses daily, consuming 100 cc 3 times a day.

Furthermore, long-term IPMC2 exceeding 1 year involved administering Jayun-tang (JYT) for exacerbated constipation. JYT was prescribed during the following periods: from Jan 05, 2023, to Jan 30, 2023; from May 20, 2023, to May 30, 2023; from Jul 19, 2023, to Jul 25, 2023; and from Aug 19, 2023, to Apr 29, 2024. Lastly, long-term IPMC3 was utilized for managing HTN with Cheonghyeol-dan (CHD). In cases where the patient’s systolic blood pressure (sBP) remained above 150 despite a head-up position for 30 minutes, 2 capsules of CHD were prescribed as needed. CHD capsules were prescribed during the following periods: from Jan 21, 2023, to Jan 29, 2023; from Feb 13, 2023, to Mar 07, 2023; from Jun 17, 2023, to Jul 03, 2023; from Oct 04, 2023, to Oct 17, 2023; from Dec 03, 2023, to Dec 21, 2023; Mar 12, 2023, and Mar 31, 2023.

HJT typically consists of 9 components, while its modified prescriptions HJT-A and HJT-B include 12 and 9 components, respectively. The composition and dosage of each prescription are detailed in Table 2. Each herbal component was weighed with an electronic scale, extracted in 1 L of water at 100°C for approximately 80 minutes using an electric decoction machine, filtered, and then aliquoted into 80 cc portions.

**Table 2:** Compositions and functions of herbal medicines prescribed for the patient.

Herb	HJT	HJT (A)	HJT (B)	Effect in TKM
*Osterici Radix* (Ganghwal)	4	4	4	Exterior-releasing
*Schizonepetae Spica* (Hyeonggae)	4	4	4	
*Saposhnikovia Radix* (Bangpung)	4	4	4	
*Angelicae Pubescentis Radix* (Dokhwal)	4	4		
*Plantaginis Semen* (Chajeonja)	4	4	4	
*Hoelen* (Baekbokryeong)	8	8	8	Urination-promoting
*Alismatis Rhizoma* (Taeksa)	8	8	4	
*Rehmanniae Radix Preparata* (Sukjihwang)	8	8		Kidney-tonifying
*Corni Fructus* (Sansuyu)	8			
*Rehmannia glutinosa Libosch* (Saengjihwang)		8–4	20	Stomach heat-clearing
*Gypsum Fibrosum* (Seokgo)		8		
*Arctii Fructus* (Ubangja)		8		
*Paeonia Suffruticosa Andrews* (Mokdanpi)		4		
*Akebia quinata* (Moktong)			20	Heat-clearing and urination-promoting
*Coptidis rhizome* (Hwangryeon)			4	

Herb			CHD	Effect in TKM
*Scutellaria raidx* (Hwanggeum), *Phellodendri Cortex* (Hwangbaek) *Gardeniae Fructus* (Chija), *Coptidis rhizome* (Hwangryeon)			0.24	Managing stroke
*Rheum palmatum* (Daehwang)			0.06	

Herb			JYT	Effect in TKM
*Cannabis Semen* (Majain)			3.83	Managing constipation
*Trichosanthis Semen* (Gwaruin), *Angelica gigas Nakai* (Danggui), *Magnolia officinalis* (Hubak), *Aucklandia lappa* (Mokhyang) *Rheum palmatum* (Daehwang), *Citrus aurantium* (Jigak), *Areca nut* (Binlang), *Prunus armeniaca* (Haengin) *Zingiber officinale* (Sengkang), *Rehmannia glutinosa Libosch* (Saengjihwang)			1.91	
*Osterici Radix* (Ganghwal), *Glycyrrhizae Radix* (Gamcho)			1.34	
*Carthamus tinctorius* (Honghwa)			0.57	

CHD = Cheonghyeol-dan, HJT = Hyeongbangjihwang-tang, JYT = Jayun-tang, TKM = traditional Korean medicine.

CHD was prepared in capsule form using 5 herbal ingredients, processed into 5000 capsules through a double reflux extraction with 80% ethanol at 120°C for 3 hours each session, followed by concentration and freeze-drying into 400 mg capsules. JYT consisted of fluid extracts from 14 different herbs, totaling 2760 portions, extracted in 500 L of distilled water at 80 to 90°C for 90 minutes, followed by filtration, low-temperature concentration, and spray drying into 6 g packets. All raw herbal materials used in these prescriptions were managed and quality-controlled by a professional pharmacist. Prior to preparation, all herbs underwent testing for heavy metals, microbial contamination, and residual pesticides.

## 3. Results

### 3.1. Safety of long-term IPMC over 1 year in an ESRD patient

#### 3.1.1. Kidney function (IPMC 1)

**Figure 2.**
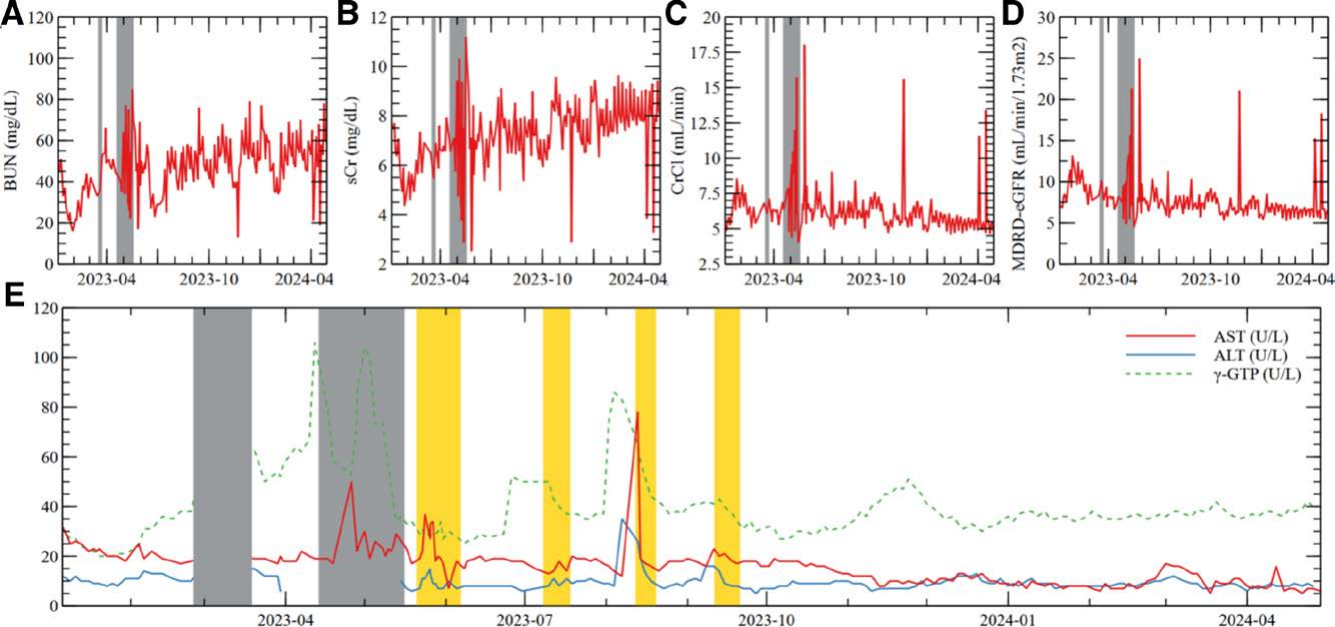
Safety indicators of long-term IPMC in an ESRD patient. (A–D) [IPMC1] Renal function trajectories; (E) [IPMC1] Liver function trajectories; (A) sCr: Variability gradually decreased, with an average sCr of 7.35 (excluding the transfer period); (B) CrCl: After the transfer period, fluctuations diminished, and the overall average CrCl stabilized around 6.20; (C) MDRD-eGFR: The average eGFR stabilized around 7.61 post-transfer period; (D) BUN: The average BUN level was 48.27; (E) AST, ALT, γ-GTP: Values remained within the normal reference range, with no increases unrelated to antibiotic treatment. Reference values: AST < 50 U/L, ALT < 50 U/L, γ-GTP 9 to 64 U/L; The values were measured at intervals of 2 to 4 d. GS transfer: March 9 to March 17, 2023; April 13 to May 16, 2023. Antibiotic treatment: May 23 to June 8, 2023; July 12 to July 25, 2023; August 13 to August 23, 2023; September 12 to September 25, 2023. γ-GTP = gamma-glutamyl transferase; ALT = alanine aminotransferase; AST = aspartate aminotransferase; BUN = blood urea nitrogen; CrCl = creatinine clearance; ESRD = end-stage renal disease; GS = general surgery; IPMC = integrative personalized medicine care; MDRD-eGFR = modification of diet in renal disease-estimated glomerular filtration rate; sCr = serum creatinine.

BUN levels (shown in Fig. 2A) during the GS transfer period ranged from a minimum of 26 mg/dL to a maximum of 85 mg/dL, with an overall average of 48.27 mg/dL. sCr (shown in Fig. 2B) levels exhibited significant fluctuations during the GS transfer period, with a minimum of 2.87 mg/dL and a maximum of 11.19 mg/dL, representing approximately a 4-fold variation. However, the variability decreased post-transfer, with sCr levels ranging from a minimum of 2.89 mg/dL to a maximum of 8.32 mg/dL until discharge. The average sCr, excluding the transfer period, was 7.35 mg/dL. CrCl (shown in Fig. 2C) demonstrated similar fluctuations during the transfer period, with a minimum of 4.02 mL/min and a maximum of 15.70 mL/min, also indicating approximately a 4-fold variation. Subsequently, the variability diminished, resulting in a concentrated distribution around an overall average CrCl of 6.20 mL/min. eGFR (shown in Fig. 2D) during the same timeframe exhibited a minimum of 4.42 mL/min and a maximum of 21.29 mL/min, reflecting approximately a 5-fold fluctuation, but later stabilized around an average eGFR of 7.61 mL/min.

#### 3.1.2. Liver function (IPMC 1)

AST, ALT, and γ-GTP levels (shown in Fig. 2E) generally remained within the reference range for patients undergoing hemodialysis, as outlined by KDIGO. On Aug 23, 2023, AST was 78 U/L, and the following γ-GTP levels were observed: 104 U/L on May 26, 2023, 100 U/L on May 27, 2023, 73 U/L on May 28, 2023, 74 U/L on May 29, 2023, 74 U/L on Sep 11, 2023, 86 U/L on Sep 13, 2023, 83 U/L on Sep 15, 2023, and 65 U/L on Sep 18, 2023. While these elevations were noted, all occurred around the time of antibiotic treatment. No elevations in AST, ALT, or γ-GTP unrelated to antibiotic therapy were observed.

### 3.2. Efficacy of long-term IPMC over 1 year in an ESRD patient

#### 3.2.1. CKD complications

##### 3.2.1.1. Malnutrition (IPMC1)

**Figure 3.**
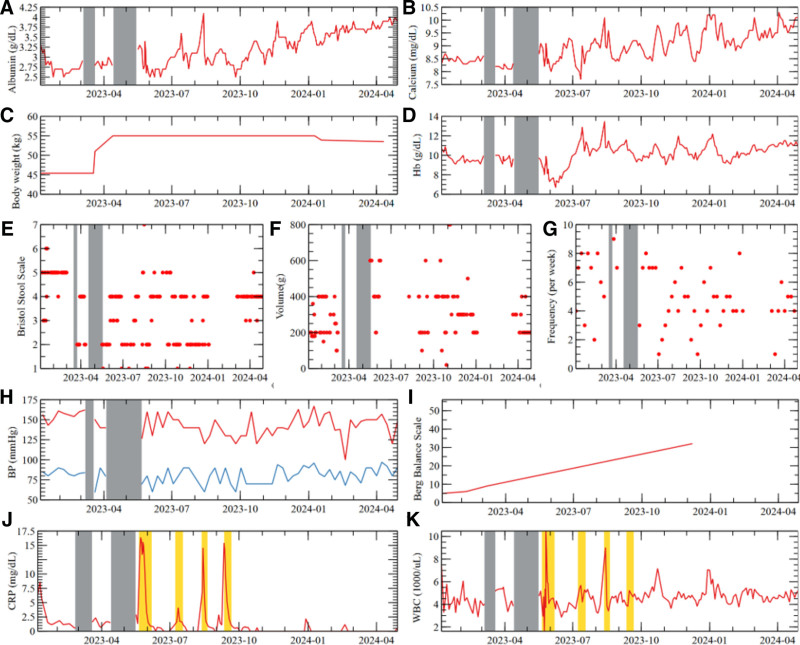
Efficacy indicators of long-term IPMC in an ESRD patient. (A–D) [IPMC1] CKD Complications: Malnutrition trajectories; (E–G) [IPMC2] CKD Complications: Constipation trajectories; (H) [IPMC3] CKD Complications: Blood pressure trajectory; (I) [IPMC4] Stroke rehabilitation trajectory; (J and K) [WM] CKD Complications: Infection trajectories; (A) Albumin: The average increased from 2.94 g/dL in the first month to 3.8 g/dL in the final month. Recommended level (KDOQI): > 4.0 g/dL^[17,18]^; (B) calcium: the average increased from 8.5 mg/dL to 9.8 mg/dL, with an overall average of 9.01 mg/dL. Recommended level (KDOQI): 8.4 to 10.2 mg/dL^[17]^; (C) body weight: increased by 9.6 kg from 45.4 kg to 55 kg, then remained stable; (D) Hb: the average increased from 7.9 g/dL in June, 2023, to 11.1 g/dL in the final month, with an overall average of 10.17 g/dL. Recommended level (KDIGO): 10.0 to 12.0 g/dL^[19]^; (E) Bristol stool scale: 98% of stool types corresponded to Types 3 and 4, indicating normal stool; (F) stool volume: average of 312 g; (G) stool frequency: the overall average was 4.9 times per week. Three weeks (4.3%) recorded 0 or 1 bowel movement per week. Gaps indicate periods with no data collected; (H) BP: the average sBP and dBP were 143 mm Hg and 80 mm Hg, respectively; (I) Berg balance scale: the score improved from 5 points at week 0 to 32 points at week 44; (J) CRP: abnormal increases were observed 4 times, but the values ultimately normalized; (K) WBC: abnormal increases were observed 4 times, but the values ultimately normalized; the values were measured at intervals of 2 to 4 days. GS transfer: March 9 to March 17, 2023; April 13 to May 16, 2023. Antibiotic treatment: May 23 to Jun 8, 2023; July 12 to July 25, 2023; August 13 to August 23, 2023; September 12 to September 25, 2023. BP = blood pressure; CKD = chronic kidney disease; CRP = C-reactive protein; dBP = diastolic blood pressure; ESRD = end-stage renal disease; GS = general surgery; Hb = hemoglobin; IPMC = integrative personalized medicine care; KDIGO: Kidney Disease: Improving Global Outcomes; KDOQI = Kidney Disease Outcomes Quality Initiative; sBP = systolic blood pressure; WBC = white blood cell.

The patient’s serum albumin level (shown in Fig. 3A) rose from an average of 2.94 g/dL in the first month of hospitalization to 3.8 g/dL in the final month. The average calcium level (shown in Fig. 3B) was 9.01 mg/dL, reflecting an increase from 8.5 mg/dL in the first month of hospitalization to 9.8 mg/dL at the time of discharge. Body weight (shown in Fig. 3C) increased from 45.4 kg by approximately 9.6 kg and was subsequently maintained at a stable level. Hemoglobin (Hb) (shown in Fig. 3D) averaged 10.17 g/dL during the hospitalization period; however, after the tunneled internal jugular power line insertion on 29. In May, 2023, the level declined significantly, averaging 7.9 g/dL in June. In contrast, the hemoglobin level increased to 11.1 g/dL by the last month of hospitalization.

##### 3.2.1.2. Constipation (IPMC2)

The Bristol Stool Scale (shown in Fig. 3E) indicated an average of Type 3 throughout the hospitalization period. Notably, since 5. In Jan, 2024, 98% of the stool types recorded – excluding 1 instance – were classified as Type 3 or Type 4, indicating normal consistency. The average stool volume (shown in Fig. 3F) during the hospitalization was 312 g. The frequency of bowel movements (shown in Fig. 3G) averaged 4.9 times per week. Weeks with fewer than 3 bowel movements, which are generally considered indicative of constipation, occurred in 6 out of 69 weeks (8.6%), of which only 3 weeks (4.3%) recorded 0 or 1 bowel movement.

##### 3.2.1.3. Hypertension (IPMC3)

The patient’s blood pressure during the hospitalization period (shown in Fig. 3H) averaged 143 mm Hg systolic and 80 mm Hg diastolic.

##### 3.2.1.4. Infection (WM)

Abnormal increases in CRP and WBC (shown in Fig. 3J and K) were observed 4 times, each requiring antibiotic treatment. On May 22, 2023, an elevated CRP level of 14.32 was detected, and from May 22, 2023 to June 6, 2023, the patient received Ceftriaxone 2 g IV every 24 hours and Meropenem 500 mg every 24 hours. On July 12,2023, with a CRP increase to 4.09, Ciprofloxacin 500 mg PO daily was administered from July 12, 2023 to July 24, 2023. On August 12, 2023, WBC rose to 8320, and the patient was treated with Meropenem 500 mg every 24 hours from August 12, 2023 to August 23, 2023. On September 11, 2023, CRP was elevated again (15.41), prompting another course of Meropenem 500 mg every 24 hours from September 12, 2023 to September 25, 2023. All antibiotics were prescribed in consultation with the division of infectious disease and discontinued once CRP and WBC levels normalized. Herbal medicines and acupuncture treatment were continued throughout the course of antibiotic therapy.

#### 3.2.2. Stroke rehabilitation (IPMC 4)

The Berg Balance Scale (shown in Fig. 3I) was assessed at week 0 (Jan, 2023, at admission), week 4 (Feb, 2023), week 8 (Mar, 2023), and week 44 (Dec, 2023). Scores improved from 5 points at week 0, to 6 points at week 4, 9 points at week 8, and 32 points at week 44.

No adverse or unanticipated events occurred during the admission.

## 4. Discussion

### 4.1. Safety of long-term IPMC over 1 year in an ESRD patient

#### 4.1.1. Kidney function (IPMC 1)

In a 10-year follow-up study conducted by Tsai et al^[20]^ to investigate the longitudinal changes in eGFR among patients with CKD, the average rate of eGFR decline in patients identified as having disease progression was reported to be 4.42 mL/min/1.73 m^2^ annually. In this case report, the MDRD-eGFR change over a period of 15 months was 1.77 mL/min/1.73 m^2^. This rate of decline is markedly slower than those previously reported in CKD patients, suggesting effective management of the progression of kidney function deterioration. Additionally, not only eGFR but also BUN, sCr, and CrCl exhibited significant fluctuations, with values differing by approximately 4 to 5 times during the initial GS transfer period. However, these parameters showed stable maintenance from that period onward until discharge. Occasional spikes in values were observed; however, considering that this patient is a dialysis patient with severe renal impairment due to ESRD and that these fluctuations were temporary occurrences, it can be said that the condition was relatively well-maintained.

#### 4.1.2. Liver function (IPMC 1)

Patients taking antibiotics, nonsteroidal anti-inflammatory drugs (NSAIDs), herbal supplements, cardiovascular drugs, and central nervous system agents are at risk of drug-induced liver injury,^[21]^ necessitating regular monitoring of liver function. In this patient, AST, ALT, and γ-GTP levels were elevated above the reference values on 9 occasions, but the degree and duration of elevation suggest a low likelihood of acute hepatitis. All elevations occurred during periods of antibiotic treatment for infections, and normalization followed the discontinuation of antibiotics, suggesting that the transient elevations were related to short-term drug-induced liver injury due to intensive antibiotic use.

As a result, this study confirms the safety of IPMC1 concerning renal and hepatic function in patients. Herbal medicine has been utilized worldwide, including in Asia, for the prevention of CKD progression and the mitigation of associated side effects. A large population-based retrospective cohort study conducted in Taiwan has demonstrated that patients with CKD receiving HM treatment experienced an increase in long-term survival rates over a 12-year follow-up period.^[22]^ However, studies confirming the safety of long-term treatment lasting over 1 year are notably limited. Thus, this study holds significant importance in demonstrating the safety of long-term IPMC treatment for over 1 year.

### 4.2. Efficacy of long-term IPMC over 1 year in an ESRD patient

#### 4.2.1. CKD complications

##### 4.2.1.1. Infection malnutrition (IPMC1)

CKD is associated with changes in appetite and taste perception, impaired thirst mechanisms, and an increased risk of dehydration. These factors contribute to malnutrition, which can lead to functional impairment, sarcopenia, and frailty, subsequently elevating the risk of falls, hospitalization rates, and mortality.^[23–29]^ Particularly in dialysis patients, concerns regarding nutritional deficiencies arise due to metabolic changes such as inadequate intake of essential nutrients from dietary restrictions, protein loss during the dialysis process, and decreased appetite, as well as psychological stress. Therefore, regular follow-up assessments are recommended starting from CKD Stage 3.^[17]^

As a result of the IPMC1 intervention, despite extremely low levels of physical activity and dietary intake, weight increased by 9.6 kg, and it has remained stable thereafter while maintaining relatively stable blood pressure. The albumin level, an important indicator of malnutrition assessment, also shows a clear upward trend on the graph, with the average level increasing from 2.94 g/dL in the first month of hospitalization to an average of 3.8 g/dL by the last month. This improvement approaches the recommended threshold of 4.0 g/dL, as suggested by the Kidney Disease Outcomes Quality Initiative (KDOQI) guidelines and supported by various studies.^[17,18]^ Anemia is linked to reduced kidney function and negatively impacts the prognosis of kidney disease. Therefore, the KDIGO guidelines recommend that this assessment be conducted at least every 3 months, especially for patients undergoing dialysis.^[30]^ In this patient, the average Hb level was recorded at 7.9 g/dL in Jun, 2023; however, a dramatic improvement was observed, with a peak Hb level of 12.9 g/dL attained in Jul, 2023, despite the absence of blood transfusions. Ultimately, by the final month of hospitalization, the average Hb level increased to 11.1 g/dL, with an overall average of 10.17 g/dL throughout the hospitalization period, thereby aligning with the KDIGO recommended range of 10.0 to 12.0 g/dL.^[19]^ Patients undergoing hemodialysis are prone to hypercalcemia, which is associated with early mortality.^[31]^ In this patient, calcium levels demonstrated a stable increase during the later stages of hospitalization, satisfying the KDOQI standards.^[17]^ This indicates that appropriate management measures have been effectively implemented.

##### 4.2.1.2. Constipation (IPMC2)

The prevalence of constipation is notably higher among patients with CKD compared to the general population, often leading to severe consequences. A study conducted by Murtagh et al reported that among the various symptoms exhibited by patients with ESRD, constipation ranks as the third most common symptom, following fatigue and pruritus, with a prevalence of 57%.^[32,33]^ Non-pharmacological treatment for constipation primarily focuses on lifestyle modifications. However, in CKD patients, concerns regarding hyperkalemia often limit fiber supplementation, and many have concurrent conditions that make it challenging to increase physical activity.

As a result of management with lactulose and HM (Jayun-tang), the average Bristol Stool Scale score during the entire hospitalization was classified as Type 3, indicating normal stool consistency. Notably, after Jan 5, 2024, approximately 98% of stool samples fell within the normal range of Types 3 and 4 on the Bristol Stool Scale, demonstrating significant improvement. The frequency of bowel movements averaged 4.9 times per week over the hospitalization period, which is considered very satisfactory. Only 6 weeks out of a total of 69 weeks met the diagnostic criteria for constipation, representing only 8.6% of the total weeks, indicating marked improvement compared to previous assessments. Lactulose was prescribed at a dosage of 60 ml per day from Jan 18, 2023 to Jan 24, 2023, 30 ml from Jan 25, 2023 to Feb 14, 2023, and 15 ml from Feb 15, 2023 to Mar 21, 2023. After Mar 21, 2023, management continued exclusively with the HM (Jayun-tang). Despite this, the most significant improvement was observed during this period, indicating that the herbal remedy effectively replaced the previous medication.

##### 4.2.1.3. Hypertension (IPMC3)

Hypertension, a crucial and modifiable risk factor for cardiovascular disease, affects over 80% of patients undergoing maintenance hemodialysis.^[34]^ A meta-analysis involving over 15,000 patients has shown that intensive treatment of hypertension in patients with CKD is associated with a reduction in mortality rates.^[35,36]^ In this case, the importance of managing hypertension is heightened, as the patient has been on oral medication for hypertension since 2007. Although the patient is undergoing dialysis and the characteristics of blood pressure indicators inherently lead to considerable variability, the implementation of IPMC facilitated the maintenance of an average systolic blood pressure (sBP) of 143 mm Hg and diastolic blood pressure (dBP) of 80 mm Hg throughout the hospitalization period, without significant deterioration. Notably, a gradual improvement in diastolic blood pressure was observed.

This paper confirms that the integrated treatment of IPMC1 to 3 was effective in managing complications such as malnutrition, constipation, and hypertension in a CKD patient. Research^[37]^ shows that herbal medicine offers benefits for CKD patients, including low cost and minimal toxicity. Zhao et al^[38]^ demonstrated that when used alongside conventional medications, herbal medicine can improve complications like hypertension and malnutrition. Its effects include anti-inflammatory, antioxidant, anti-apoptotic, autophagy-regulating, and anti-fibrotic properties. This approach may be particularly useful for patients with a frail physique, such as the 1 described (170 cm, 45.4 kg). With poor appetite and limited physical activity due to prolonged bed rest, conventional interventions like exercise are not feasible. In such cases, combining IPMC with herbal medicine, which enhances overall vitality, could be highly effective.

##### 4.2.1.4. Infection (WM)

Infection is a common cause of hospitalization in patients with ESRD, especially those undergoing hemodialysis. According to the Hemodialysis (HEMO) Study, a real-time randomized trial involving 1846 hemodialysis patients with a mean follow-up of 2.8 years, there were 1698 infection-related hospitalizations, and the annual infection incidence was 35%. The most common cause of infection was an unknown source, including sepsis, bacteremia, and abscesses unrelated to organ-specific infections. The second most common cause was vascular access-related infection, accounting for 23% of all cases. Among patients who died during the HEMO Study, infection was the leading cause of death, accounting for 23% of total deaths.^[39]^

The patient exhibited abnormal elevations in CRP and WBC on 4 occasions, each of which normalized following the administration of antibiotics. During the first episode (May 23, 2023–June 8, 2023), although blood cultures revealed no abnormal findings, bacteria were detected in sputum cultures, suggesting a potential diagnosis of pneumonia. During the second episode (July 12, 2023–July 25, 2023), the rise in CRP could have been related to catheter reinsertion or suspected pneumonia. In both the third (August 13, 2023–August 23, 2023) and fourth episodes (September 12, 2023–September 25, 2023), neither blood nor sputum cultures yielded bacterial growth, raising the possibility of an AVG site infection, although the precise cause of the infection could not be determined. Nevertheless, all episodes of antibiotic treatment were completed within 2 weeks without residual complications, indicating that both the prevention and postinfection treatment were appropriately managed.

#### 4.2.2. Stroke rehabilitation (IPMC 4)

Acupuncture, when combined with conventional treatment for poststroke rehabilitation, has been shown to have beneficial effects on improving hemiparesis,^[40]^ spasticity,^[21]^ dysphagia,^[41]^ neuralgia,^[42]^ and cognitive impairment.^[40]^ As a result, the integrated approach of combining Western treatment with acupuncture is recommended in various countries. According to research by Birch and Robinson, out of 84 clinical practice guidelines and treatment guidelines from 27 countries related to stroke aftercare and rehabilitation, 49 clinical practice guidelines and treatment guidelines from 35 countries positively recommended acupuncture.^[43]^ These findings support the efficacy of acupuncture in stroke management, as also evidenced by improvements in the patient’s Berg Balance Scale scores. Although the Berg Balance Scale was not assessed regularly in this patient, with only 4 evaluations performed, the consistent improvement observed in each assessment suggests that stroke rehabilitation was being conducted appropriately.

### 4.3. Significance and limitations of the study

This case report investigated the effects and safety of administering personalized herbal medicine based on IPMC alongside conventional standard treatment and acupuncture to a patient with ESRD over a period exceeding 1 year. The results demonstrated stable maintenance of sCr, eGFR, and BUN levels compared to pre-dialysis values, suggesting the potential effectiveness of herbal medicine in stabilizing renal function in patients with chronic renal failure. These findings are consistent with the potential renal protective effects of herbal medicine reported by Wang et al^[6]^, which are believed to originate from the inhibition of inflammatory responses and prevention of renal fibrosis.^[44]^ Additionally, no herbal medicine-related adverse effects were observed during the study, and the patient’s liver function markers (AST, ALT, γ-GTP) remained within normal ranges, indicating the safe function of the herbal treatments. Thus, long-term concurrent administration of herbal and conventional medicine can be considered effective and safe for patients with ESRD.

However, this study has several limitations. First, the safety of long-term IPMC regarding nephrotoxicity in dialysis patients cannot be fully confirmed based solely on laboratory markers. Second, minor elevations in liver function markers were observed during the hospitalization period, which were temporary, occurred exclusively during antibiotic administration, and did not indicate severe complications. Third, several unexplained abnormal increases in CRP and WBC levels were noted, necessitating further investigation in future studies. Additionally, the frequency of assessments using the Berg Balance Scale for stroke rehabilitation was limited, and more comprehensive tools, such as the Modified Barthel Index or NIHSS, would have provided a more robust evaluation of poststroke hemiparesis. Finally, as this is a single-patient case report, the generalizability of the findings is inherently restricted.

Despite these limitations, the IPMC approach, which emphasizes personalized care tailored to individual patient characteristics, holds significant promise. This case report establishes a foundation for future investigations into the efficacy and safety of herbal medicine-based IPMC. The findings suggest that personalized therapy may represent a transformative paradigm in chronic disease management.

Building upon these findings, future research should pursue several key directions. First, long-term cohort studies with follow-up periods exceeding 5 years are necessary to comprehensively evaluate the potential nephrotoxicity of IPMC in patients with ESRD. These studies should monitor not only conventional renal function indicators (sCr, eGFR, BUN), but also incorporate sensitive renal injury biomarkers such as kidney injury molecule-1 (KIM-1) and neutrophil gelatinase-associated lipocalin (NGAL) to provide a more detailed safety profile. Second, comparative clinical studies involving both dialysis and non-dialysis patient groups would help clarify the differential effects and synergistic potential of combining herbal and standard therapies, thereby expanding the clinical applicability of IPMC. Third, pharmacological studies using in vivo and in vitro models are needed to clarify the mechanisms of action of the herbal formulations used in IPMC. These investigations may help elucidate the renal protective effects of specific herbal components at the molecular level. Collectively, these efforts will help establish a robust evidence base for the safe and effective integration of personalized herbal medicine into standard CKD management.

## 5. Conclusion

In conclusion, this study demonstrated that the long-term concurrent administration of herbal medicine prescriptions based on IPMC with standard drugs is safely feasible for patients with ESRD. The results also suggest that combining herbal and acupuncture treatments with standard care may contribute to the improvement of complications and long-term quality of life in ESRD patients.

Although this is a retrospective single-patient case report, the patient and caregiver expressed satisfaction with the treatment during hospitalization, and the IPMC approach has been continued through outpatient care. These clinical observations suggest the potential of IPMC as a supportive strategy in CKD management.

To strengthen the evidence base, future research should include long-term cohort studies assessing nephrotoxicity, comparative trials between dialysis and non-dialysis patients, and pharmacological investigations to clarify the mechanisms of action of key herbal components.

## Acknowledgments

This study was supported by a grant of the Korea Health Technology R&D Project through the Korea Health Industry Development Institute (KHIDI), funded by the Ministry of Health & Welfare, Republic of Korea (grant number: RS-2024-00441603).

**Statement of Ethics**Study approval statement: This study protocol was reviewed and approved by the Institutional Review Board of Kyung Hee University Korean Medicine Hospital, approval number [2024-10-020]. Consent to publish statement: Not applicable

**Conflict of Interest Statement**The authors have no conflicts of interest to declare.

**Funding Sources**The funder had no role in the design, data collection, data analysis, or reporting of this study.

**Disclaimer**The views expressed in this article are those of the authors and do not necessarily reflect the official policy or position of their affiliated institutions or the journal.

## Author contributions

**Conceptualization:** Eui-Ju Lee.

**Data curation:** Hyeon-Ji Yu.

**Funding acquisition:** Eui-Ju Lee.

**Investigation:** Chae-Yeon Kang, Ju-Yeon Lim, Eui-Ju Lee, Yu-Na Lee, Won-Hee Choi, Hyeon-Ji Yu.

**Methodology:** Chae-Yeon Kang, Ju-Yeon Lim, Eui-Ju Lee, Yu-Na Lee, Won-Hee Choi, Hyeon-Ji Yu.

**Project administration:** Eui-Ju Lee.

**Visualization:** Chae-Yeon Kang, Ju-Yeon Lim.

**Writing – original draft:** Chae-Yeon Kang, Ju-Yeon Lim, Yu-Na Lee, Won-Hee Choi.

**Writing – review & editing:** Eui-Ju Lee.
